# Knowledge of final year medicine, pharmacy, audiology and nursing students in South Africa on drug-induced ototoxicity: A pilot study

**DOI:** 10.4102/sajcd.v66i1.606

**Published:** 2019-11-14

**Authors:** Omphile Mogole, Natalie Schellack, Cara Hollander, Lebogang Ramma

**Affiliations:** 1 Department of Clinical Pharmacy, School of Pharmacy, Sefako Makgatho Health Sciences University, Pretoria, South Africa; 2 Department of Speech Pathology and Audiology, University of the Witwatersrand, Johannesburg, South Africa; 3 Department of Health and Rehabilitation Sciences, University of Cape Town, Cape Town, South Africa

**Keywords:** Multidisciplinary, ototoxicity, training, pharmacotherapy, students

## Abstract

**Background:**

There is a high prevalence of human immunodeficiency virus (HIV), tuberculosis (TB), cancer and malaria in South Africa, and the drugs used to treat these conditions can be ototoxic. It is therefore important that healthcare professionals are able to identify and understand these drugs and their effects to ensure effective care of the patient.

**Objective:**

This study aimed to assess the knowledge regarding pharmacotherapy-induced ototoxicity amongst final year, medicine, pharmacy, audiology and nursing students across South African universities.

**Methods:**

A descriptive cross-sectional study design was used, and data were collected via a self-administered online questionnaire. Non-probability purposive sampling was used to identify the participants at the universities which train audiologists, pharmacists, medical and nursing students.

**Results:**

An overall response rate of 41% (*n* = 720) was obtained. Sixty-four per cent (*n* = 461) of respondents were women (median age: 23 years). The majority of the respondents (95%) knew what pharmacotherapy-induced ototoxicity was, but a few (39%) knew the general signs and symptoms of ototoxicity. Furthermore, just less than half of the sample (48%) could identify the specific ototoxic medicines and the type of damage caused by this medication.

**Conclusion:**

To manage pharmacotherapy-induced ototoxicity effectively, a multidisciplinary healthcare team must have sufficient knowledge about ototoxicity. Therefore, efforts should be made to introduce extensively concepts of pharmacotherapy-induced ototoxicity into the undergraduate curricula of pharmacy, medical, nursing and audiology programmes.

## Introduction

According to Schellack, Wium, Ehlert, Van Aswegen and Gous (2015), pharmacotherapy-induced ototoxicity is a growing issue, especially in developing countries such as South Africa. The prevalence of tuberculosis (TB), human immunodeficiency virus (HIV) and malaria in South Africa predisposes these patients to hearing loss which is induced by agents used to treat these conditions (Schellack et al., 2015). While there are no real statistics regarding the incidence of hearing loss because of HIV and malaria, Petersen and Rogers (2015) estimate that up to 90% of patients with TB, specifically drug-resistant TB, may experience hearing loss. Neonates, particularly those born prematurely, are more prone to infections because of their immunocompromised nature. Furthermore, the antibiotics, for example, amikacin, that are used to treat these infections have been shown to be ototoxic in approximately 59% of these babies (Engler, Schellack, Naude, & Gous, 2013).

Furthermore, Schellack et al. (2015) state that pharmacists along with medical professionals and nurses play an integral role in identifying and monitoring ototoxic agents, while audiologists often get involved only after the patients have completed therapy and start experiencing hearing loss. Early identification of hearing loss and balance problems (ototoxicity monitoring) is of paramount importance to prevent or minimise permanent impairment and therefore improve the quality of life post-treatment (Saindane, 2016). Thus, audiologists need to be involved at the initial stages of patient care and part of the multidisciplinary team.

To assist this multidisciplinary care, interprofessional education (IPE) is needed. According to Treadwell and Havenga (2013), IPE refers to the learning from and between the disciplines of various health science students in order to improve collaboration and the quality of patient care. Interprofessional education is an important approach to develop health science students, as trained students are more likely to become collaborative interprofessional team members who show respect and positive attitudes towards each other and work towards improving patient outcomes (Bridges, Davidson, Odegard, Maki, & Tomkowiak, 2011). However, medicine, pharmacy, nursing and other health professions are supposed to work together in a clinical environment, which requires effective teamwork and communication skills. The majority of university-based health professional education currently delivers a discipline-specific model of teaching (Lapkin, Levett-Jones, & Gilligan, 2013).

Furthermore, a relationship between the physician (as the prescribers) and nurses (as the care takers) needs to be established. This relationship should be supported by education regarding the importance and benefits of drug monitoring and as well as the evaluation of possible adverse effects, which should be discussed with the patients (Konrad-Martin, Wilmington, Gordon, Reavis, & Fausti, 2005). According to Vasquez and Mattucci (2003), many physicians do not understand the importance of otolaryngologists and audiologists in pre-treatment counselling and evaluation and the need for follow-up assessments of the patient’s auditory function.

A multidisciplinary team approach, particularly between physicians, pharmacists, audiologists and nurses, is integral to collaborative patient education programmes regarding ototoxic medication, its effects and management. This is considering that most primary care clinicians and clinical pharmacists, particularly in developing countries such as South Africa, have seen patients who have suffered from ototoxicity at some point in their careers (Schellack et al., 2015). Hearing loss, especially when occurring during childhood, can negatively impact communication development, scholastic performance and quality of life (Phanguphangu & Ramma, 2018). Therefore, identification and understanding of the drugs and their impacts can improve patient care and outcomes. However, literature pertaining to the core curriculum or knowledge and perceptions of these healthcare workers in training is limited, especially in the South African context.

## Method

### Study design, setting and population

A descriptive cross-sectional survey design was used. The target population for this study included all final year pharmacy, medical, audiology and nursing students across South African universities (see Table 1). Non-probability purposive sampling was used to select the participants, as this type of purposive sampling is able to produce a sample considered as ‘representative’ of the population (Battaglia, 2008).

**TABLE 1:** Universities offering pharmacy, medical, audiology and nursing degrees.

University	Pharmacy	Medical	Nursing	Audiology
Nelson Mandela Metropolitan University	X	*	X	*
University of Forte Hare	*	*	X	*
Walter Sisulu University	*	*	X	*
University of Free State	*	*	X	*
Rhodes University	X	*	*	*
Tshwane University of Technology	X	*	*	*
University of Limpopo (Turfloop Campus)	X	*	*	*
University of the Western Cape	X	*	*	*
Sefako Makgatho Health Sciences University	X	X	X	X
University of Pretoria	*	*	*	*
University of the Witwatersrand	X	X	*	X
Vaal University of Technology	*	*	X	*
Durban University of Technology	*	*	X	*
University of KwaZulu-Natal	X	*	*	*
University of Cape Town	*	*	*	X

* , Departments or schools that did not participate in the study; X, universities that participated in the study.

### Data collection instruments and procedures

A randomised, self-developed questionnaire (Appendix 1) was the primary data collection tool in this study. The questionnaire was developed and compiled using information from an article by Schellack and Naude (2013) regarding pharmacotherapy-induced ototoxicity. The questionnaire explored the following themes of knowledge in relation to pharmacotherapy-induced ototoxicity: general knowledge, prevention, ototoxicity monitoring and the prescription of ototoxic drugs.

Data collection was conducted over a period of 24 weeks. A link to the questionnaire was sent to a designated staff member, who was either the head of department or someone chosen by the head of department. This person was the one who sent out the link of the study to the students in the department and was responsible for disseminating the link to the final year students in their respective departments. Implied consent was used. Reminder emails pertaining to the online questionnaire were sent to these designated staff members at the university departments or schools on a weekly basis throughout the data collection period. The aim of this communication was for the designated staff members to remind students to complete and submit the questionnaire on the online platform.

### Data analysis

Data were entered into Microsoft Excel® spreadsheets and exported to IBM Statistical Analysis Software® (SAS) for analysis. Prior to analysis, universities were de-identified and recoded as ‘A’, ‘B’, ‘C’, ‘D’, ‘E’, ‘F’, ‘G’, ‘H’, ‘I’, ‘J’, ‘K’, ‘L’ and ‘M’.

Frequencies and percentages were calculated for all variables, including knowledge in relation to pharmacotherapy-induced ototoxicity. This encompassed general knowledge, prevention, ototoxicity monitoring and the prescription of ototoxic drugs.

Fisher’s exact test was used to test for differences in response and the Chi-square test was used to test for associations in responses between the different universities. Statistical significance was set at *p* ≤ 0.05.

### Ethical consideration

Ethical approval was sought from the University Research Ethics Committees before commencement of data collection (SMUREC/H/246/2016: PG). Permission for the study was obtained from the head of each department and research ethics committees at the different universities. Implied consent was used for the purposes of the study and has been stated as part of the data collection instrument. The completion of the questionnaire implied consent to participate in the study. Participation was voluntary, and the responses were anonymous.

## Results

### Response rate

Questionnaires were sent to all universities that train medicine, pharmacy, nursing and audiology students; therefore, an estimated 1760 potential participants were expected to take part in this study. Seven hundred and twenty final year students from all courses across South African universities participated in the study. This figure represents an average response rate of only 41% (25% – 86% across the different universities and courses). The response rates by institution and course are shown in Table 2.

**TABLE 2:** Response rate by university and course.

University	Number (*n*) and response rate (%)							
	Pharmacy (*N* = 266)		Medical (*N* = 210)		Nursing (*N* = 188)		Audiology (*N* = 56)	
	*n*	%	*n*	%	*n*	%	*n*	%
A	*	*	*	*	23	26	*	*
B	46	39	*	*	42	46	*	*
C	35	25	*	*	*		*	*
D	49	77	11	27	34	85	19	86
E	*	*	*	*	*	*	13	43
F	*	*	*	*	30	32	*	*
G	*	*	*	*	27	49	*	*
H	38	44	*	*	*	*	*	*
I	31	48	*	*	*	*	*	*
J	39	39	*	*	*	*	*	*
K	28	46	98	31	*	*	24	67
L	*	*	*	*	14	58	*	*
M	*	*	*	*	24	47	*	*

* , Departments or schools that did not participate in the study.

### Demographics

Overall, 64.03% of the respondents were women. Over two-thirds (68.19%) of the students were between the ages of 22–25 years; 6.94% were younger than 22 years and 24.72% were over the age of 25 years.

### General knowledge of pharmacotherapy-induced ototoxicity

Respondents’ general knowledge of pharmacotherapy-induced ototoxicity is summarised in Table 3. A vast majority of the respondents (95%) demonstrated knowledge about ototoxicity; however, only a small proportion of respondents (28%) identified the signs of ototoxicity (*p* < 0.001), and 21% of the respondents knew the risk factors of pharmacotherapy-induced ototoxicity (*p* < 0.001).

**TABLE 3:** General knowledge of final year pharmacy, medical, nursing and audiology students regarding pharmacotherapy-induced ototoxicity.

Knowledge area	Students per university (%)					*p*
	All	Pharmacy (*n* = 266)	Medical (*n* = 210)	Nursing (*n* = 188)	Audiology (*n* = 56)	
What is ototoxicity?	95	97	94	95	93	0.091†
Signs of ototoxicity	28	39	35	18	21	< 0.001†
Patients are at risk of developing ototoxicity	21	23	18	24	20	< 0.001‡

† , Chi-square test;

‡ , Fisher’s exact test.

### Drugs causing ototoxicity

Respondents’ knowledge regarding drugs causing ototoxicity is summarised in Table 4. Forty-eight per cent of participants identified ototoxic drugs, with pharmacy students performing slightly better than the other groups. Only 36% of the respondents knew how non-steroidal anti-inflammatory drugs (NSAID)-induced ototoxicity occurred (*p* < 0.001) and 43% of the respondents accurately reported that hearing loss caused by aminoglycosides was irreversible, with significant differences between knowledge of students in the different disciplines (*p* < 0.001). Close to one-fifth of the respondents (19%) identified ototoxic anti-hypertensive medication (*p* < 0.001). Forty-three per cent were able to identify ototoxic anti-TB medication, with significant difference in responses between the different courses (*p* < 0.001).

**TABLE 4:** Final year pharmacy, medical, nursing and audiology students’ knowledge about drugs that cause ototoxicity (percentage of students who answered correctly).

Knowledge area	Students per university (%)					*p*
	All	Pharmacy (*n* = 266)	Medical (*n* = 210)	Nursing (*n* = 188)	Audiology (*n* = 56)	
Drugs that do not cause ototoxicity	48	62	53	44	32	< 0.001†
How does NSAID-induced ototoxicity occur?	36	35	35	40	34	< 0.001‡
Which TB medication causes hearing loss?	43	47	40	44	41	0.063‡
Which anti-hypertensives are likely to cause hearing loss?	19	23	23	14	14	< 0.001†
Is aminolgycoside-induced ototoxicity reversible?	43	35	53	42	41	< 0.001‡

NSAID, non-steroidal anti-inflammatory drugs.

† , Chi-square test;

‡ , Fisher’s exact test.

### Type of damage caused

Respondents’ knowledge regarding the type of damage caused by ototoxic drugs is summarised in Table 5. A quarter of the respondents (25%) knew the ototoxic side effects caused by aspirin (*p* < 0.001). Just over 50% of the medical students (52%) identified the most ototoxic medicine from a list of ototoxic medications (*p* < 0.001). Pharmacy students showed poor knowledge regarding the signs of damage to the cochlea and vestibular system. Pharmacy (42%) and audiology students (43%) showed a fair knowledge of the type of hearing loss caused by cisplatin and vancomycin, with both pharmacy (13%) and audiology (27%) also showing insufficient knowledge regarding the cause of sensorineural hearing loss (*p* < 0.001).

**TABLE 5:** Final year pharmacy, medical, nursing and audiology students’ knowledge about the type of damage caused by ototoxic medicine (percentage of students who answered correctly).

Knowledge area	Students per university (%)				*p*
	Pharmacy (*n* = 266)	Medical (*n* = 210)	Nursing (*n* = 188)	Audiology (*n* = 56)	
What are the ototoxic effects of aspirin?	25	26	14	36	< 0.001†
Which drugs are most ototoxic?	*	52	*	*	< 0.001†
What are the side effects of damage to the vestibular organ?	25	*	*	*	< 0.001†
Drugs that cause reversible hearing loss (erythromycin) do not have to be monitored for ototoxic effects	34	*	*	*	< 0.001†
Which frequencies does pharmacotherapy-induced hearing loss affect?	47	*	*	*	< 0.001†
What are the side effects of damage to the cochlea?	21	*	*	*	< 0.001‡
Which hair cells are most susceptible to ototoxic damage?	23	*	*	45	0.530†
Cisplatin and vancomycin cause which type of hearing loss?	42	*	*	43	0.041†
High-frequency sensorineural hearing loss is caused by damage to which structure?	13	*	*	27	0.234†

* , Departments or schools that did not participate in the study.

† , Chi-square test;

‡ , Fisher’s exact test.

### Discipline-specific questions

Discipline-specific questions were aimed at assessing what the students needed to know regarding pharmacotherapy-induced ototoxicity within their specific disciplines, namely, pharmacy, medical, nursing and audiology. Respondent’s discipline-specific knowledge is summarised in Table 6. Less than half of the respondents (45%) knew the steps which needed to be taken when a patient starts experiencing ototoxic side effects. Just over half of the pharmacy students (54%) knew the otoprotective anti-oxidant properties of N-acetylcysteine (*p* < 0.001), while 23% knew the strategies required to minimise drug-induced ototoxicity. The majority of both pharmacy (61%) and audiology (75%) students understood what otoacoustic emissions were. Fourteen per cent of pharmacy students and 29% of audiology students knew what pure tone audiometry tests are used for in determining hearing loss. Both pharmacy (36%) and audiology (39%) students showed slight knowledge regarding when baseline audiometric tests should be performed for patients taking ototoxic medication. Pharmacy students seemed to have a better knowledge regarding when audiometric monitoring should be initiated (46%) than audiology students did (30%). Just over half of the medical students (54%) reported that a patient should not receive more than one ototoxic medicine at a time, with only 31% of them being able to identify what to do in the event of ototoxicity.

**TABLE 6:** Discipline-specific knowledge of final year pharmacy, medical, nursing and audiology students regarding pharmacotherapy-induced ototoxicity.

Knowledge area	Students per university (%)				*p*
	Pharmacy (*n* = 266)	Medical (*n* = 210)	Nursing (*n* = 188)	Audiology (*n* = 56)	
As a healthcare professional, what should you do when you suspect that a patient may be showing some ototoxic signs/symptoms?	42	55	36	46	0.058†
N-acetylcycsteine can be used as a protective agent in patients treated with ototoxic drugs	54	*	*	*	< 0.001‡
What are the strategies used to minimise ototoxicity?	23	*	*	*	< 0.001‡
Otoacoustic emissions are sounds generated by the cochlea’s sensory hair cells in response to auditory stimulation	61	*	*	75	0.049‡
What are otoacoustic emissions used for?	16	*	*	14	< 0.001‡
Baseline testing should be performed ___ treatment has started	36	*	*	39	0.037†
What are pure tone audiometry tests used for?	14	*	*	29	0.01‡
Monitoring of ototoxicity can only be done once the patient starts experiencing ototoxic effects	46	*	*	30	0.250‡
Pharmacotherapy-induced hearing loss affects which frequencies?	47	*	*	64	0.757†
A patient can receive more than one ototoxic drug as long as the doses are not high	*	54	*	*	0.067‡
What should be done if a patient starts experiencing ototoxic side effects?	*	31	*	*	< 0.001†
When prescribing ototoxic drugs, the risk-to-benefit ratio should be taken into account	*	66	*	*	< 0.001†
Patients experiencing ototoxic effects should be referred for audiological monitoring only after the treatment has finished	*	52	*	*	< 0.001†
Which of the following drugs can be used concurrently with ototoxic medicine to try and minimise the ototoxic effects?	*	11	*	*	< 0.001†
When should audiological monitoring be initiated?	*	26	*	*	< 0.001†
Patients receiving ototoxic drugs should not be counselled about the possible side effects as this will prevent treatment	*	35	*	*	0.166‡
Most ototoxic drugs do not cause hearing loss when given at therapeutic doses	*	59	*	*	< 0.001‡
Patients with a past history of hearing loss should not take any ototoxic drugs	33	60	*	*	< 0.001‡

* , Departments or schools that did not participate in the study.

† , Chi-square test;

‡ , Fisher’s exact test.

Just over 60% of medical students indicated that the risk-to-benefit ratio should also be considered when prescribing ototoxic medication, with significant differences between the individual universities (*p* < 0.001). Just over half of the medical students (52%) indicated that patients who experience ototoxic effects should be referred for audiological monitoring only once the therapy has been completed (*p* < 0.001). A few of the medical students (35%) reported that patients should be counselled before treatment about the possible ototoxic side effects of the medicine and 59% reported that most ototoxic medicine will not cause hearing loss when given at therapeutic doses. Only 33% of pharmacy students indicated that patients with a past history of hearing loss should not take ototoxic drugs, and 60% of medical students agreed with them (*p* < 0.001).

## Discussion

This study evaluated final year pharmacy, medical, nursing and audiology students’ knowledge regarding pharmacotherapy-induced ototoxicity. To the best of our knowledge, this study is the first to assess these aspects amongst pharmacy, medical, nursing and audiology students across South African universities. This study attempted to assess all South African universities; however, not all chose to participate in the study.

The results indicated that the majority of the students across different universities and professional fields of studies may not have adequate basic knowledge regarding pharmacotherapy-induced ototoxicity, as determined by the questionnaire in this study. Across all universities, the students were able only to define ototoxicity, but could not identify the signs of ototoxicity and risk factors involved. The results are also in agreement with those reported by Khoza-Shangase and Jina (2013), a study which revealed that doctors do not have adequate knowledge of ototoxicity symptoms and treatment. Pharmacy students, however, scored much better (97%) than students from other courses in terms of general knowledge regarding pharmacotherapy-induced ototoxicity, which may be attributed to the fact that they have more extensive pharmacology training at undergraduate level than the other courses. However, as they should take an active role in the identification of ototoxic drugs, and the potential management, a greater knowledge is still required.

Furthermore, most of the respondents could not adequately identify drugs that cause ototoxicity. Audiology students scored the lowest for this question (32%), which can be explained by the fact that they do not have pharmacology training at undergraduate level. This supported a study conducted by Naidoo (2006) in which the majority of the audiologists were of the opinion that their undergraduate clinical training had left them unprepared to implement pharmacotherapy-induced ototoxicity monitoring services. This could indicate that perhaps the curriculum needs to be adapted to include pharmacology for audiologists. The respondents’ knowledge regarding the type of damage caused by ototoxic medicine was shown to be poor. Respondents could not adequately identify whether certain medicines caused reversible or irreversible hearing loss. Also, the respondents could not determine the signs that arise because of damage to a specific structure of the ear (cochlear and vestibular system) as well as which drugs cause damage to which structure.

With respect to discipline-specific questions, pharmacy and medical students performed relatively well in terms of identifying the steps to follow if a patient starts experiencing ototoxicity and preventative measures that can be taken, including the pre-treatment that can be used when a patient is receiving ototoxic medicine, as compared to the other disciplines such as nursing and audiology. This is in line with an article by Lapkin et al. (2013), who described a report by the World Health Organization (WHO, 2016) which suggested that a large percentage of hearing impairment was attributed to ototoxic medication as a result of inappropriate use by healthcare providers, most especially from those prescribing. Knowledge was inadequate regarding when audiological monitoring should be initiated, and also regarding prevention and pre-treatment of pharmacotherapy-induced ototoxicity. Nursing students showed poor knowledge in terms of steps to be taken when a patient starts experiencing ototoxic side effects. Audiology students scored acceptably with regard to the steps that need to be followed when patients start experiencing ototoxic side effects and showed slight knowledge regarding when audiological monitoring should be initiated, as the majority of them indicated that audiological monitoring can be done only once a patient has completed treatment.

A strength of the study was that it was conducted amongst final year undergraduate students at a point when they had completed most of their formal academic curriculum to ensure that their knowledge would be assessed close to completion of their degrees. The study also had some limitations; use of an online survey might have reduced the response rate and difficulty permission from some universities. Also, not all universities that train these professionals participated in the study. The reasons for the lack of participation was either because the relevant head of department or dean did not respond to the numerous emails that were sent out or declined to participate in the study.

## Conclusion

The study demonstrated that final year pharmacy, medical, nursing and audiology students across South African universities lack sufficient knowledge regarding pharmacotherapy-induced ototoxicity. The gaps in knowledge regarding the topic indicate that there may be insufficient undergraduate training pertaining to ototoxicity. The results suggest that pharmacy, medical, nursing and audiology students should be adequately trained at undergraduate level to handle cases of pharmacotherapy-induced ototoxicity once they get into the professional multidisciplinary healthcare environment to ensure patient safety. This would include the identification of potentially ototoxic medication, counselling of patients, and further ongoing monitoring and management of the drug and hearing loss. Schellack and Naude (2013) highlighted the importance of a coordinated and cooperative healthcare team in the early detection of cochleotoxicity, which holds especially true for a country such as South Africa that has a high disease burden, which often includes treatment by potentially ototoxic medication.

## Appendix 1

Questionnaire on pharmacotherapy-induced ototoxicity.

Dear Candidate,

Please choose the sections applicable to you.

### Section A: Demographics

**(To be completed by everyone)**

Please tick the appropriate box or give the necessary information in the space provided.

**Table:**

**Gender:**	
Male	
Female	

**Table:**

**Age:**	
18–21 years	
22–25 years	
26–30 years	
30+ years	

**Which course are you currently doing?**

**Table:**

Pharmacy	
Medicine	
Audiology	
Nursing	

**Which university are you enrolled at?**

**Table:**

University	Mark with ‘x’
Nelson Mandela Metropolitan University (NMMU)	
• University of Forte Hare (UFH)	
• Walter Sisulu University	
• University of Free State (UFS)	
• Rhodes University	
• University of Limpopo (Turfloop Campus)	
• University of the Western Cape (UWC)	
• Sefako Makhatho Health Sciences University (SMU)	
• University of the Witwatersrand (WITS)	
• Vaal University of Technology (VUT)	
• Durban University of Technology (DUT)	
• University of KwaZulu-Natal (UKZN)	
• University of Cape Town (UCT)	

### Section B: General knowledge regarding pharmacotherapy-induced ototoxicity

**(To be completed by everyone)**

1. Ototoxicity is
  - A side effect of medicine resulting in auditory and/or vestibular dysfunction resulting in hearing loss and disequilibrium
  - A toxic reaction resulting from an interaction between two or more drugs
  - Renal impairment because of medicine overdose
  - A side effect of medicine resulting in severe skin rash
  - I don’t know
2. Signs of ototoxicity include:
  - Hearing loss
  - Disequilibrium
  - Renal impairment
  - A and B
  - A, B and C
  - I don’t know
3. Which of the following drugs does not cause ototoxicity?
  - Vancomycin
  - Cisplatin
  - Penicillin
  - I don’t know
4. Which patients are at risk of developing ototoxicity?
  - Patients with renal impairment
  - MDR-TB patients
  - Heart failure patients
  - All of the above
  - I don’t know
5. Aminoglycosides-induced ototoxicity is reversible.
  - True
  - False
  - I don’t know
6. NSAID-induced ototoxicity may occurs when
  - Patients use the medication for a prolonged period
  - Double the normal dose is administered
  - Patients take a normal dose for a few days
  - All of the above
  - I don’t know
7. Aspirin causes the following ototoxic effects:
  - Hearing loss
  - Tinnitus
  - Disequilibrium
  - Earache
  - All of the above
  - I don’t know
8. Which medicine used for TB is likely to cause hearing loss?
  - Rifampicin
  - Ethambutol
  - Amikacin
  - Isoniazid
  - None of the above
  - I don’t know
9. Hypertensive patients are at risk of ototoxicity when using high doses of which anti-hypertensive
  - Hydrochlorothiazide
  - Enalapril
  - Lorsatan
  - Furosemide
  - I don’t know
10. As a nurse, when you suspect that a patient may be showing some ototoxic signs/symptoms, you must:
  - Observe the patient for a while before referring them
  - Refer to an audiologist for confirmation
  - Inform the treating physician
  - Wait until the end of the treatment before referring the patient
  - I don’t know

### Section C: Prevention of pharmacotherapy-induced ototoxicity

**(To be answered by pharmacy students)**

1. N-acetylcysteine (ACC-200®) can be used as a protective agent in patients treated with ototoxic drugs
  - True
  - False
  - I don’t know
2. Which of the following is the most correct preventative measure for patients receiving ototoxic medicine?
  - Change medication
  - Counsel patient
  - Change dosage
  - All of the above
  - I don’t know
3. The protective anti-oxidant effects of which drug is related to its action as a free radical scavenger?
  - Paracetamol
  - N-acetycsteine
  - Sodium chloride
  - Adrenaline
  - None of the above
  - I don’t know
4. Patients can receive more than one ototoxic drug as long as the doses are not too high.
  - True
  - False
  - I don’t know
5. Damage to the vestibular organ results in which side effects?
  - Tinnitus
  - Hearing loss
  - Dizziness
  - Loss of balance (disequilibrium)
  - A and B
  - C and D
  - I don’t know
6. Patients with a past history of hearing loss should not take any ototoxic medicine.
  - True
  - False
  - I don’t know
7. Which of the following patients are at a higher risk of aminoglycoside ototoxicity?
  - Those taking therapy for more than 5 days
  - Those taking higher doses
  - Elderly patients with renal insufficiency
  - All of the above
  - I don’t know
8. Drugs that cause reversible hearing loss (erythromycin) do not have to be monitored for ototoxic effects.
  - True
  - False
  - I don’t know
9. Strategies to minimise ototoxicity include:
  - Keep patients hydrated
  - Dosage instructions should be strictly followed
  - Noisy environments should be avoided for at least 6 months after treatment
  - A and B
  - A, B and C
  - I don’t know

### Section D: Monitoring of pharmacotherapy-induced ototoxicity

**(To be completed by audiology and pharmacy students)**

1. Otoacoustic emissions are sounds generated by the cochlea’s sensory hair cells in response to auditory stimulation.
  - True
  - False
  - I don’t know
2. Pharmacotherapy-induced hearing loss affects
  - Lower frequencies first and then progresses to the higher frequencies
  - Higher frequencies first and then progresses to the lower frequencies
  - Both lower and higher frequencies at the same time
  - I don’t know
3. Otoacoustic emissions are used to
  - Monitor effects of treatment
  - Treat ototoxicity before it progresses
  - Determine which medicine caused the ototoxicity
  - Change the sound generated by the cochlea
  - I don’t know
4. Damage to the cochlea results in which side effects?
  - Tinnitus
  - Hearing loss
  - Dizziness
  - Loss of balance (disequilibrium)
  - A and B
  - C and D
  - I don’t know
5. Which hair cells are more susceptible to ototoxic damage?
  - Inner hair cells
  - Outer hair cells
  - Middle hair cells
  - All of the above
  - I don’t know
6. Cisplatin and vancomycin causes which type of hearing loss?
  - Reversible
  - Irreversible
  - I don’t know
7. High-frequency sensorineural hearing loss is caused by damage to the
  - Cochlea
  - Vestibular organ
  - Tympanic membrane
  - Ear drum
  - I don’t know
8. Baseline testing should be performed _________ treatment has started
  - 2 h before
  - 24 h before
  - 2 h after
  - 24 h after
  - I don’t know
9. Pure tone audiometry tests determine
  - Absence or presence of hearing loss
  - Extent of hearing loss
  - The cause of hearing loss
  - All of the above
  - I don’t know
10. Monitoring of ototoxicity can be done only once the patient starts experiencing ototoxic effects (e.g. tinnitus).
  - True
  - False
  - I don’t know

### Section E: Prescribing of ototoxic medicine

**(To be completed by medicine students)**

1. A patient can receive more than two ototoxic medicines as long as the doses are not high.
  - True
  - False
  - I don’t know
2. If a patient starts experiencing ototoxic effects, the following should be done:
  - Change the medicine to less toxic ones
  - Counsel the patient
  - Change the dose of the medicine
  - All of the above
  - I don’t know
3. When prescribing ototoxic drugs, the risk-to-benefit ratio should always be taken into consideration.
  - True
  - False
  - I don’t know
4. Patients experiencing ototoxic effects should be referred for audiological monitoring only after the treatment has finished.
  - True
  - False
  - I don’t know
5. Which of the following drugs can be used concurrently with ototoxic medicine to try to minimise the ototoxic effects?
  - Adrenaline
  - N-acetycysteine
  - Sodium chloride
  - Paracetamol
  - None of the above
  - I don’t know
6. Audiological monitoring should be initiated
  - Before treatment, during treatment and after treatment
  - Only before treatment to assess hearing
  - Only after treatment to assess effect of treatment
  - Only during course of treatment
  - None of the above
  - I don’t know
7. Patients receiving ototoxic drugs should not be counselled about the possible effects as this will prevent treatment compliance.
  - True
  - False
  - I don’t know
8. Most ototoxic drugs do not cause hearing loss when given at therapeutic doses.
  - True
  - False
  - I don’t know
9. Which of the following drugs are the most ototoxic?
  - Furosemide
  - Erythromycin
  - Vancomycin
  - Aspirin
  - I don’t know
10. Patients with a past history of hearing loss should not take any ototoxic medicine.
  - True
  - False
  - I don’t know
